# Genome engineering and plant breeding: impact on trait discovery and development

**DOI:** 10.1007/s00299-016-1993-z

**Published:** 2016-05-18

**Authors:** Fabien Nogué, Kostlend Mara, Cécile Collonnier, Josep M. Casacuberta

**Affiliations:** INRA AgroParisTech, IJPB, UMR 1318, INRA Centre de Versailles, Route de Saint Cyr, 78026 Versailles Cedex, France; Center for Research in Agricultural Genomics, CRAG (CSIC-IRTA-UAB-UB), Campus UAB, Cerdanyola del Vallès, 08193 Barcelona, Spain

**Keywords:** Genome engineering, Plant breeding, Genetic diversity, Site-directed nucleases, QTLs

## Abstract

*****Key message***:**

**New tools for the precise modification of crops genes are now available for the engineering of new ideotypes. A future challenge in this emerging field of genome engineering is to develop efficient methods for allele mining.**

**Abstract:**

Genome engineering tools are now available in plants, including major crops, to modify in a predictable manner a given gene. These new techniques have a tremendous potential for a spectacular acceleration of the plant breeding process. Here, we discuss how genetic diversity has always been the raw material for breeders and how they have always taken advantage of the best available science to use, and when possible, increase, this genetic diversity. We will present why the advent of these new techniques gives to the breeders extremely powerful tools for crop breeding, but also why this will require the breeders and researchers to characterize the genes underlying this genetic diversity more precisely. Tackling these challenges should permit the engineering of optimized alleles assortments in an unprecedented and controlled way.

## Genetic diversity, mutations and plant breeding: a fruitful “ménage à trois”

In spite of the remarkable fidelity of DNA replication and the high efficiency of genome repair and surveillance systems, mutations are incorporated into the DNA at a rate that makes virtually every organism unique. In plants, the mutation rate has been determined to be of 7 × 10^−9^ per site and generation in *Arabidopsis thaliana* (Ossowski et al. 2010), which means that even for this very compact genome, roughly one mutation is incorporated in each newborn individual. These mutations, which in some cases trigger phenotypic differences among individuals, are the raw material on which selection can operate making species adaptation and long-term evolution possible.

The most frequent types of genomic mutations are short insertions/deletions and single nucleotide changes leading to single nucleotide polymorphisms (SNPs). However, genomes also experience many drastic changes, including chromosome rearrangements, long deletions, duplications and insertions of long sequences, such as transposable elements. In fact, it is considered that the two major mechanisms for plant genome dynamics are the whole genome doubling (WGD) events (Wendel 2015) and the differential and lineage-specific proliferation (and deletion) of various types of transposable elements (TEs) (Lisch 2013). All these mechanisms endow plant genomes with a remarkable plasticity that allows plants to adapt to new environments and that has also been exploited for their domestication and breeding. The domestication of wild species and their breeding are a particular type of evolution directed mainly by human selection. However, the principles and mechanisms governing these processes are by no means different from those involved in general evolution. In fact, domestication and breeding constitute excellent models to study evolution at large (Meyer and Purugganan 2013; Olsen and Wendel 2013).

### Domestication and crop breeding alleles

During the last 10 years, an impressive amount of plant domestication and improvement alleles have been characterized and we can now start to pinpoint the genes and the mutations underlying the substantial phenotypic differences between crops and their wild relatives. There are a number of traits that have been repeatedly selected during plant domestication. Essentially, these are traits that facilitate crop planting and harvesting and are collectively referred to as the domestication syndrome (Olsen and Wendel 2013). A list of the traits already characterized can be found in recent reviews (Meyer and Purugganan 2013; Olsen and Wendel 2013). These include loss of seed shattering and seed dormancy, uniformity in germination and erect growth allowing increased plant density in crop fields. Although the same traits have been repeatedly selected in domestication processes, it seems that the genes behind the phenotypic changes were not always the same, suggesting that this adaptive process is remarkably flexible (Gaut 2015). This is the case of the dual domestication of common bean, for which a population genetics analysis showed that among the genes presenting signs of having been selected, those that are common in both events are not different from a random expectation (Schmutz et al. 2014).

On the other hand, it is also becoming increasingly clear that many domestication-related traits are not monogenic but involve many genes with small effects (Olsen and Wendel 2013).

However, even taking into account the complexity and the flexibility of the genomic changes associated to crop domestication, the analysis of the many relatively simple examples already characterized allows for deducing general trends of this process. In addition, these analyses provided us with a collection of genes and specific alleles behind important phenotypic transitions that can be of use for plant breeding. As already noticed in 2006 (Doebley et al. 2006), changes in morphology or other complex phenotypes often involve transcriptional or development regulators, and rarely structural proteins and enzymes (Olsen and Wendel 2013).

### Mutations behind the domestication and breeding phenotypic changes

The most frequent causal mutations of the domestication phenotypes are nonsense mutations, such as premature truncation of ORFs through induced frameshifts, introduction of STOP codons, changes in splicing signals, or AA changes leading to a loss of function of the protein. For example, the transition from the prostrate growth of ancestral wild rice to the erect growth of rice cultivars, which was one of the critical events in rice domestication, was the result of the selection of a single mutation in the *PROG1* (*PROSTRATE GROWTH 1*) gene. This mutation produces an AA substitution in the zinc-finger nuclear transcription factor encoded by *PROG1* leading to the loss of function (Jin et al. 2008; Tan et al. 2008).

However, mutations inducing changes in cis-regulatory sequences or changes in AAs leading to modified activity are also relatively frequent. For example, the key domestication change on plant architecture in maize (i.e., loss of auxiliary branches and increased apical dominance) was due to a mutation affecting the expression of the *tb1* (*teosinte branched 1*) gene (Wang et al. 1999). In this case, the causal mutation has been shown to be the insertion of a *Hopscotch* retrotransposon, some 60 kb upstream of the coding region, that increases *tb1* expression (Studer et al. 2011). *tb1* encodes a transcription regulator that represses growth and its overexpression in the auxiliary branches inhibits their development. As for mutations that change the function of a protein, an interesting example is that of the *Flowering Locus T* (*FT*) paralogs in sunflower that played a role in the domestication of this species. It has been shown that a frameshift mutation in the *Helianthus annuus FT 1* (*HaFT1*) gene generates a dominant-negative allele that interacts and blocks the action of another FT paralog resulting in delayed flowering (Blackman et al. 2010).

In addition to traits that can be considered as linked to the domestication process, crops have also been intensively modified in other traits to improve their cultivation and the quality and the diversity of their derived products. Here again, spontaneous mutations through the panoply of already mentioned mechanisms caused null alleles but also modified the expression or the coding capacity of genes. An interesting example that illustrates the high plasticity of plant genomes is the mutation associated to the *SUN* gene, linked to the elongated phenotype of tomato, which consists of a retrotransposon-mediated gene duplication that placed the duplicated gene under the control of a different promoter in the genome (Xiao et al. 2008).

In summary, plant domestication and breeding led to the profound modification of plant phenotypes through the recurrent selection of spontaneous mutations present in plant populations. These mutations encompass a diversity of genome changes, from SNPs and small indels to chromosomal rearrangements, gene duplications and TE insertion/deletion events that have modified an important number of genes, often regulators of key processes in the plant.

### Introducing new mutations

Plant breeding has been done for the most part of its 10,000 years of history in an unconscious way. However, the progress of the scientific knowledge has an immediate impact in these practices throughout history. Understanding the key role of genetic variability and selection had a profound impact on plant breeders and the discovery that physical and chemical agents induce mutations in the DNA (Muller 1928; Stadler 1928) prompted the breeders to start mutagenesis programs to increase genetic variability. Over the past 70 years, more than 3200 new crop varieties in over 200 plant species have been obtained through mutagenesis (IAEA/FAO mutant variety database, http://www-infocris.iaea.org/MVD/). This approach has proved to be extremely useful despite its limitations (Parry et al. 2009). Mutations introduced by these methods are of the same type as spontaneous mutations. Most mutations consist of SNPs and short indels but long insertion/deletions, chromosome rearrangements and gene duplications are also frequent (Bolon et al. 2014).

The most important limitation of chemical and physical mutagenesis is its stochastic nature. Generating mutants for specific traits requires the generation of extensive mutant collections and the development of powerful forward genetic screens. A refinement of the strategy of induced mutagenesis is TILLING (which stands for Targeting Induced Local Lesions In Genomes) that links mutagenesis to a DNA-analysis method that selects from thousands of mutagenized plants those that display the mutations in the desired gene. However, in most cases, the outcome of these approaches is null mutations and obtaining particular alleles known to confer certain phenotypes is, in most cases, virtually impossible.

In the last few years, a new generation of mutagenic agents, based on site-directed nucleases (SDNs) [or site-specific nucleases (SSNs)], such as zinc-finger nucleases, TALENs or the more recent CRISPR/Cas9, has been developed and very rapidly been incorporated into the plant breeders toolkit (Podevin et al. 2013). SDNs allow the generation of all the types of mutations that have been introduced during the domestication and breeding of crops with an unprecedented precision and extent. Different genes can be the targets of these SDNs, and interesting alleles can be discovered through forward and reverse genetic screens, for simple traits. However, for more complex traits, the use of these new techniques will require the identification of the genomic regions involved by the quantitative traits loci (QTL) analysis.

## Genetic diversity captured by breeders through the identification of QTL

### New techniques for the discovery of QTLs

Quantitative traits loci mapping has so far been very efficient for identifying the genetic regions linked to quantitative agronomic traits. It was supported by the recent development of new means of high-throughput genotyping and phenotyping. However, it is still a challenge to identify genomic regions associated to phenotypes that could result from different stresses and to show overlapping responses in plants. This is particularly the case for abiotic stresses, where repeated evaluations to confirm the value of the detected QTLs are needed (Takeda and Matsuoka 2008). Moreover, QTL mapping is limited to the detection of genetic variants in populations segregated from bi-parental crosses. Some other techniques that are now used in routine for the detection of alleles of interest, such as association mapping and selection screening, have been developed (Takeda and Matsuoka 2008). Association mapping correlates genetic markers to a given phenotype based on the analysis of populations derived from large germplasms. It allows for a large number of alleles for each tested locus to be taken into account, instead of the two parental alleles tested with classical QTL mapping (Takeda and Matsuoka 2008). A key factor in this approach is the linkage disequilibrium (LD) that determines the marker density and the experimental design. For species, such as *A. thaliana* and maize, that exhibit a high recombination rate and a rapid decay of LD (<1 kb), this approach is successful (Yu and Buckler 2006). In self-crossing crops, such as rice, soybean, sorghum and barley, the relatively large scale of LD leads to a lower association mapping resolution. An alternative approach, selection screening, is based on the theory that, after strong selection, the selected loci present a decrease in nucleotide diversity and an increase in LD (Takeda and Matsuoka 2008). This approach is not suitable for all crops, as for sorghum, for example, where no selection was found after the screening of 445 loci (Hamblin et al. 2006). On the contrary, it is particularly well suited for species, such as maize, for which the demographic history has been well studied. However, the choice between the different approaches by the breeders will influence the strategy and the success rate of their breeding plans.

### Exploitation of the QTLs necessitates long and cost effective breeding plans

In the last two decades, numerous efforts have been made to identify molecular markers and QTLs linked to important agronomic traits for a wide range of crop species. Marker assisted selection (MAS) is one of the most used techniques to select for QTLs in various plant species. An alternative approach that has found wide application is known as QTL pyramiding. Here, combinations of two or more QTLs identified in different varieties or compatible species and responsible for different traits are introgressed into the same elite line (Ashikari and Matsuoka 2006). However, contrasting results were reported regarding the successful rate of the QTL introgression, especially when five or more QTLs for certain traits are introgressed into a given elite line (Semagn et al. 2006). Among the various factors that negatively impact the achievement of successful results of MAS, the low repeatability of QTLs through different genetic backgrounds, interactions between QTLs, and recombination between genes that might be present in the same chromosomal segment within the QTL are the most reported ones (Semagn et al. 2010). Another limit of working with QTLs is that their exploitation requires long and expensive breeding plans. This is particularly true when working with forest tree species. Indeed, most forest trees are clonally propagated resulting in narrow genetic diversity and small segregating populations. In addition, they have large genomes, grow under highly heterogenic environmental conditions and, most importantly, their generation cycles are very long (Lidder and Sonnino 2012). Technological advances, such as genome editing, will provide new perspectives for more efficient breeding strategies.

### Characterization of the genes underlying QTLs could be an exceptional source of targets for genome engineering

Once QTLs involved in a desired trait have been identified unequivocally, all the genes present in those loci need to be characterized to look for polymorphic genes linked to the desired phenotype. When an annotated version of the whole genome sequence is available, the putative function of the genes might point towards particular ones (candidate genes). This approach is more complicated for species, whose genomes are still not available, or for genes, whose function is not known. The availability and continuing cost decrease of molecular markers and the development of new genomic techniques, such as NGS, might enhance efficiency in identifying and characterizing candidate genes for various species (Pérez-de-Castro et al. 2012; Lim et al. 2014). The validation of the candidate gene is the key step of the whole process. For that matter, one possible strategy consists of the analysis of the genetic profile of the candidate gene with the aim of finding some nucleotide variations (i.e., SNPs, premature stop codons, etc.) that are absent in another variety of the same species which does not present that phenotype/trait. In the case, the comparison with other varieties of the same species is not possible, and a study of the importance of those particular mutations can be carried out in a model plant, where the presence of a reference genome and availability of obtaining mutants are more feasible than in other plant species. For this purpose, genetic engineering techniques can help to introduce point mutations. Once the link of that particular nucleotidic variation with the wanted phenotype/trait is certified, the same kind of variation can be introduced in the desired variety of interest or elite line. Direct modification of the variety of interest/elite line genome through genetic engineering techniques can result in restoring the desired trait and reducing the time of introgression of the allele by avoiding backcrossing of a large number of inbred lines. In the conventional breeding, a minimum of five-six backcrossing generations is necessary to transfer the gene of interest from a donor to a recipient line (Lidder and Sonnino 2012). This process requires a lot of time and effort, especially when more than one allele needs to be transferred.

### Role of model plants in the validation of the candidate genes

The use of model plants, with a well-characterized genome, and ample genetic and molecular resources, is of high value in the validation of candidate genes for interesting phenotypes. The confirmation of the role of candidate genes in model species is a good indicator of their potential implication in the related crop species. For the final validation in crops, again, academic laboratories could provide assistance to breeders for the rapid production of the desired genetic profiles by genome engineering. Academic labs would benefit from their implication in such studies by deepening their overall knowledge of crop genomics and enlarging the field of application of their discoveries. Thus, cooperation between breeders/farmers and public research institutions could be very fruitful for the genome engineering of elite lines and should be enhanced by appropriate innovation programs.

## Showcasing of the candidate genes through genome engineering of elite lines

### Precise modification of elite line genomes

Once an allele responsible for a particular desired trait has been identified, its transfer to an agronomically interesting variety can sometimes be a challenge. For that matter, genome engineering can provide means of inserting or modifying it into elite lines. Because of the new SDNs capable of inducing mutagenesis or gene replacement at specific targets, new alleles can be engineered very precisely at endogenous loci and with a very limited impact on the rest of the genome. Different types of mutations (deletions, insertions, and substitutions) can be generated randomly via error-prone non-homologous end-joining (NHEJ). In addition, it seems that by targeting a locus presenting micro-homologies, the output of the repair, that could be mediated via micro-homology mediated end-joining (MMEJ), can be predicted (Butler et al. 2015; Collonnier et al. 2016).

In addition to the introduction of targeted mutations in a particular gene, SDNs can be used to induce mutagenesis in different genes simultaneously, which can be particularly interesting for the modification of loci underlying quantitative traits. For that matter, several SDNs targeting different loci can be co-transformed (Ma et al. 2015; Shan et al. 2015; Lowder et al. 2015), but a single SDN targeting a sequence common to a defined set of genes can also be used. This latter approach is very useful to knock-out TAGs (tandemly arrayed genes) which are prevalent in higher eukaryotic genomes (e.g., ~17 % in *A. thaliana*) and to trigger gene clusters deletions (Christian et al. 2013; Qi et al. 2013; Zhou et al. 2014). Multiplexing can also lead to chromosome rearrangements and create novel chimeric genes (Qi et al. 2013). Inducing multi-KOs is particularly interesting for the creation of new phenotypes depending on paralogous genes in diploid species or on multiple homeologous genes in polyploid species (Wang et al. 2014). Thus, SDNs are particularly amenable to engineer complex plant genomes and to confer valuable traits to crops with high ploidy and genome size (Gil-Humanes and Voytas 2014).

### Knock-out and knock-in strategies

Knock-out of specific candidate genes through imprecise NHEJ-mediated repair of SDN-induced DSBs offers a lot of opportunities for the creation of valuable agronomic traits in crops. Some innovative processing traits were developed in potato lines using TALENs targeting the three alleles of the *VInv* gene (vacuolar invertase). The resulting mutations limit the accumulation of reducing sugars in tubers during cold storage and minimize the production of acrylamide during frying (Clasen et al. 2016). Different quality traits were also produced via the knock-out strategy. Fragrant rice was created by targeted knock-out of the *OsBADH2* gene using TALENs (Shan et al. 2015). Tomatoes with a modified fruit ripening pattern were produced by CRISPR/Cas9-mediated mutagenesis of the *RIN* locus (Ito et al. 2015). Soybean lines with improved oil quality were obtained by TALEN-induced targeted mutagenesis of the fatty acid desaturase 2 gene families (Haun et al. 2014). Knocking-out host susceptibility genes is also a good way to develop a new resistance to diseases. In bread wheat, TALEN-induced mutation in three homeologous genes (*TaMLO*) conferred resistance to powdery mildew (Wang et al. 2014). TALENs and CRISPRs were also used to engineer resistance to bacterial blight in rice by targeting the binding sites of bacterial transcription activator-like effectors (Li et al. 2012; Zhou et al. 2015a). Provided that candidate genes are identified, and other traits, such as tolerance to abiotic stresses (drought), resistance to diseases (such as sharka in *Prunus*) or better nutritional quality (lesser anti-nutritional compounds and healthier fatty acids) might be within reach via the knock-out strategy. However, knock-in of new alleles into the genome by homologous recombination-mediated targeted gene replacement offers an even wider range of potentialities and will probably be the method of choice in the future. So far, very few agronomic traits have been engineered by gene targeting in crops. Resistance to herbicides was, for example, conferred to tobacco lines by ZFN-stimulated allele replacement of two endogenous genes (Townsend et al. 2009). One technical way of supporting the soaring of the knock-in strategy could be to develop methodologies facilitating the proper and stable integration of the donor template. New tools, such as the recently identified nucleases Cpf1, which trigger sticky ends DSBs instead of blunt cuts like the Cas9 protein (Fagerlund et al. 2015), have to be explored.

### Will genome editing progressively take over marker assisted selection (MAS)?

The association of defined regions of the genome to traits of interest relies on diagnostic markers identified through the detection of classical QTLs by bi-parental crossing, or via association mapping. In MAS, these markers are then used to predict the genetic value of the individuals that are kept in the successive generations of breeding (Das and Rao 2015; Hasan et al. 2015; Pradhan et al. 2015). As proposed before, genome editing could help to speed up the breeding process of QTLs normally followed by MAS technology. Provided the number and the size of the genomic regions associated with the desired traits are not too high, one could even imagine that, in a near future, it could replace MAS in some cases. Because of the possibility of targeting different loci at the same time, the modifications could be done over very few generations, depending on the total number of loci to target (the number of SDNs capable of working simultaneously in a given cell being high but not unlimited) (Farré et al. 2014) and on the type of transformation used (some leading to homogenous and stable transformants in T0, T1 or T2 generation) (Feng et al. 2014). There is then no need then for labor intensive, costly and time-consuming backcrossing to perform QTL pyramiding. This approach is particularly indicated for mono- or oligo-genic traits and for polygenic traits depending on few genes with strong effects. For quantitative traits depending on many genes with small effects, whether the candidate genes are transferred into elite lines by hybridization or by genome engineering, the value of the phenotype may not be exactly the one expected, partly due to the modification of genetic backgrounds and the resulting loss of the relevant genetic interactions (Takeda and Matsuoka 2008). Genome editing could be very useful to evaluate and validate the strength of the predictive value of a given candidate gene by easily transferring its best alleles into a set of different genetic backgrounds representative of the diversity of the genetic material used in the selection schemes. As multiple genes can be individually engineered at the same time, genome editing also provides a way to modify linked genes or QTLs that are usually difficult to segregate due to the limits of meiotic recombination (Flavell 2010).

### Beyond the constraints of elitism: increasing transformation and mutation frequencies

The promise of totipotency in the context of transformation has not yet been fulfilled in all crops. Genetic modifications were preferably realized in varieties amenable to transformation and regeneration protocols, that were, once transformed, backcrossed to elite lines. The optimization and the industrialization of certain critical steps of these protocols now allow for the acceleration the breeding process for many crops by directly transforming elite lines. For these crops, candidate genes can be engineered in elite lines without difficulty. For the others, genome editing relies on the development of robust and possibly genotype-independent methods of transformation and regeneration adapted to these highly selected materials. Most of the time, regeneration is the bottleneck and a better understanding of the genetics of this trait would be determinant. For the moment, more immediate solutions consist in trying to increase transformation efficiency and mutation frequency. One promising way is to use engineered plant virus expression vectors, such as modified geminivirus replicons, to express SDNs and bring the donor DNA (Butler et al. 2015; Čermák et al. 2015; Yin et al. 2015; Baltes et al. 2015). This approach also presents the advantage to produce edited plants without stable integration of the SDNs-coding transgenes that are only transiently expressed and do not need to be segregated in the progeny (Čermák et al. 2015). It is particularly interesting for species with high degrees of heterozygosis and long juvenile stages, such as fruit trees, for which genetic segregation would be long and costly (Ilardi and Tavazza 2015). This applies also to vegetatively propagated species, such as potato or grapes, for which segregation has a strong genetic cost. Very efficient mutation frequencies were also obtained in certain species by transforming nucleases directly as proteins (Luo et al. 2015; Woo et al. 2015). Finally, mutation efficiency can be optimized using specific promoters favoring the expression of SDN-coding genes in reproductive cells, the ratio of mutated T1 plants being sometimes 20-fold higher than with constitutive promoters (Wang et al. 2015).

### No-one left behind: breeding to support development

Genome editing could contribute to support the breeding of orphan crops that are of key economic importance in certain local environments, in particular in developing countries. These crops regroup species, such as sorghum and millets, groundnut, cowpea, common bean, chickpea, pigeonpea, cassava, yam and sweet potato (Varshney et al. 2012). They do not benefit from large investments from both public research institutes and private breeding companies. As a consequence, very little genomic information is available on them, which makes difficult the mapping of regions involved in traits of interest, as well as the testing of candidate genes. MAS can also be complicated by the use of landraces or wild germplasm as the source of favorable alleles (Varshney et al. 2012). By allowing very precise modification of genomic regions and targeted introgression of potential candidate genes coming from any source, genome editing can help lift some of these difficulties. In addition, by making breeding of new varieties less labor intensive and less costly, it could not only facilitate the work of local research teams, but also make private investment into small programs dedicated to this type of crops more appealing.

Several initiatives aimed at developing GM traits to answer health and agronomic issues in developing countries have been conducted in the world. They have been long tested without full success due to societal and governmental opposition to the arrival onto the market of seeds modified via classical transgenesis. The Golden Rice case (Potrykus 2015) and the attempts to grow virus-resistant papaya in Asia (Davidson 2012; Gonsalves 2015) illustrate very well this problem.

### Back to the future: looking for candidate genes in wild species

As presented before, the search for the QTLs and identification of the genes that are beyond is a major source of information for the development of SDNs strategies. Most of the time, the choice of the material used for the QTLs identification is driven by the capacity of the parent lines to be cross-fertile with elite lines that will be the final recipient of the allele(s) of interest. In general, the genetic bottlenecks imposed on crops during domestication and through modern breeding practices have greatly reduced the genetic variability that can be used for breeding. For this reason, wild relatives or local landraces are a particularly useful source for allele mining (Tanksley and McCouch 1997). As a consequence, there are now more and more reports proposing to look for genetic diversity in wild species or local landraces through selection screens (Zhou et al. 2015b). However, crosses are not always easy or even feasible between domesticated varieties and their wild relatives. This barrier of sexual compatibility limits the gene pools accessible to the breeders (Michelmore 2003). On the contrary, in principle, the sole limitation for the application of the SDNs technology to a specific gene is the presence of its homolog in a donor organism, which could be a cross-incompatible wild relative or even more distant species, including non-plant species (e.g., bacteria). In addition, SDNs can also be used for what some authors have called a “rewilding” of actual crops through GE to get rid of mutations that compromise the hereditary basis of crop survival during environmental stresses and are rarely counter-selected (Palmgren et al. 2015). Thus, the search for superior alleles of a given agronomically important gene should no longer be restricted any more to related genotypes but should be extended to divergent ones.

## Genome tuning through SDNs to go a step further than classical plant breeding

### Inventing new candidate genes

As already explained, the search for new alleles for plant improvement is the basis of plant breeding. However, this search for superior alleles should not be restricted to natural variations but should also include artificial design or induced genetic variations leading to the discovery of alleles otherwise not present in the available genetic diversity. Indeed, in addition to the screening of natural variation, several technologies can be employed for the improvement of a gene product, including protein-structure-based rational design (Lutz 2011), random mutagenesis and the more recent strategy of directed evolution. Directed evolution of proteins integrate random and focused mutagenesis using different strategies, such as error-prone PCR, DNA shuffling (in vivo or in vitro), and screening for better alleles in microbe systems (Minshull and Stemmer 2001). These strategies have been very useful for improving or altering the activity of biomolecules for industrial, research and therapeutic applications (Packer and Liu 2015) and, although they are still in their infancy for plant proteins (Lassner and Bedbrook 2001), they have a great potential for the optimization of some agronomically important gene products. However, we must keep in mind that these screenings in microbial systems will be essentially valid for enzymes and that the optimization achieved in a bacterial system will not necessarily be maintained in the plant. One way to avoid these two limitations would be to implement the directed evolution strategy in the plant itself.

### Targeted induced genetic variation (TIGV) an alternative to TILLING?

**Fig. 1:**
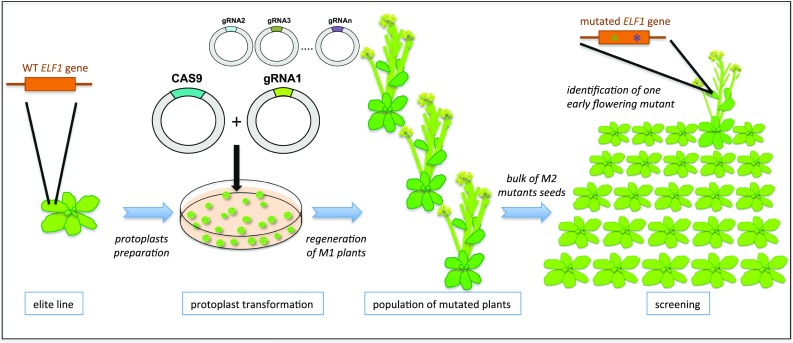
Principle of targeted induced genetic variation (TIGV). The aim of TIGV is to produce genetic variability at one or more loci in the genome and screen for individuals that would have acquired, at the targeted loci, more favorable alleles for one or more agronomic traits. In this case, the desired trait is a short-cycle rapeseed line. For this purpose, the *ELF1* gene that has been shown to be involved in the transition from the vegetative to reproductive growth will be targeted using the CRISPR/Cas9 strategy. Wild type plants are stably or transiently (in this case via protoplasts PEG fusion) transformed with the CAS9 nuclease and one or more gRNAs targeting different sites in the *ELF1* gene. M1 plants are regenerated and selfed. M2 mutants seeds are then collected and sown in field conditions for screening of early flowering plants

Mutations, in combination with recombination, are the sources from which plant breeders are able to produce new varieties. As stated before, one way to increase the genetic variability available for breeding is to induce mutations and characterize them using the TILLING technique. However, the use of the newly produced allele in breeding plans is still time consuming and necessitates multiple cycles of crossing for introgression in an elite line. One way to avoid or limit these time-consuming steps of introgression could be the use of SDNs for Targeted Induced Genetic Variation (TIGV) (Fig. 1). CRISPR/Cas9, and in general SDNs, can be used to introduce specific or random mutations at a precise location in the genome depending on the use or not of a template DNA to repair the introduced DSB. In addition, a collection of SDNs can be engineered to introduce a combination of DSBs in the same gene. In the case of CRISPR/Cas9, this can be easily achieved using a collection of guide RNAs instead of a single one. This approach could be used to mutate a gene in multiple sites also presenting different modifications, allowing to generate a population of alleles of a particular gene. This would strongly increase the chance to find, for a given gene product, a superior allele that would result from a combination of specific mutations in multiple domains of the protein. One obvious target for TIGV is resistance genes, such as the NBS-LRR gene family, as the evolution of these genes is thought to be facilitated by recombination and sequence exchange, leading to variation in the recognition patterns of plants to pathogen elicitors (Joshi and Nayak 2013). Last but not least, the use of TIGV on different genes in the same plant would permit the generation of a population of mutants that would contain different combinations of alleles at different loci. Strategies consisting in the targeting of multiple genes in the same plant (multiplex editing) have been proven functional in crops (Xie et al. 2015). The production of such targeted mutagenized populations and their screening in different conditions of stresses would represent a very innovative solution to tackle the question of multiple and combined stresses in crop production (Kissoudis et al. 2014). Moreover, because stress conditions, such as salinity and drought, vary depending on local climates and geographical features, site-specific screening of these populations of mutants should permit the selection of superior gene alleles for very specific agricultural conditions leading to custom-made breeding. Nevertheless, the TIGV strategy has limitations. The first one is the capacity to transform efficiently elite lines for a given crop. Moreover, as the strategy is based on screening for mutants in natural conditions of growth, a second limitation is the possibility to grow stably transformed GM plants (i.e., mutated plants where the CAS9/gRNA module has not been segregated) in field trials.

### “Genome remodeling”, crop genome optimization through SDNs

The use of SDNs is not restricted to the modification of coding sequences but can also be used to delete large chromosomal segments (up to 170 kb) or target repeated sequences, as already demonstrated using the CRISPR/Cas9 strategy in rice, for example, (Endo et al. 2015; Zhou et al. 2014). As already commented, these types of genomic structure-associated mutations, such as insertions, deletions, duplications, inversions and translocations, are part of the genomic differences within a species that are not limited to single nucleotide. These large polymorphisms are named structural variants (SVs) (Saxena et al. 2014). Some SVs have been clearly associated with specific phenotypes, such as aluminum-tolerance in maize that is due to an increase in the copy number of the *MATE1* gene (Maron et al. 2013). However, the SVs are not always associated with a gain of function and some of them can be detrimental to fitness. In addition, the proliferation of repetitive sequences can also be detrimental to the genome. For example, the banana genome is invaded by numerous badnavirus sequences, including those of banana streak viruses (BSVs) and some of these named endogenous BSVs (eBSVs), can release infectious pararetrovirus following stresses (Chabannes et al. 2013). Potential awakening of eBSVs has become the major constraint to banana breeding programs worldwide (Chabannes et al. 2013). One way to get rid of this risk could be the specific knock-out or, even better, the deletion of the BSVs sequences in the banana genome using the CRISPR/Cas9 strategy.

### Mimic the CRISPR/Cas9 defense system in plants

Some of these new tools of genome engineering can also be used for purposes other than modifying the plant genome. The CRISPR-Cas system originates from bacteria and is an adaptive immune system to fight against foreign DNA, such as phages and, in some aspects, could be compared to the eukaryotic, RNA interference (RNAi) pathway, even if these two systems are not homologous (Marraffini and Sontheimer 2010). Recently, different groups have tried to transpose this bacterial immune system to plants. For this purpose, the CAS9 nuclease and single guide RNA targeting different sequences in the genome of geminiviruses have been expressed in *Nicotiana benthamiana* and Arabidopsis (Ali et al. 2015; Baltes et al. 2015; Ji et al. 2015) and shown to induce mutations in the viral DNA sequence and to decrease the copy number of replicating virus. Interestingly, the co-expression of two sgRNAs in the plant increases the efficiency of reduction of viral genome copy number and targeting a common sequence in different viruses allows multiple virus resistance (Ali et al. 2015; Baltes et al. 2015). Thus, this strategy, due to its flexibility and ease of implementation, presents a great potential to protect plants against viruses, although this needs to be confirmed in field conditions. Moreover, like all the strategies to fight against viruses, the CRISPR/Cas9 also has potential limitations. First, because resistant virus strains could emerge due to mutations appearing in the targeted sequences and preventing the cleavage, or due to the production by the virus of suppressors of the CRISPR/Cas9 system, as it is already the case for some bacteriophages (van der Oost and Brouns 2015). Second, the constitutive expression of a CAS9/sgRNA module into the plant raises the question of the potential risk of unintended mutagenic effects (due to direct or off-target interaction) on the plant genome and/or on organisms that would be in contact with the plant.

### CRISPR/Cas9 and friends to control meiotic recombination

The improvement of crop species relies on the possibility to produce new allele combinations, and, as mentioned before, new techniques, such as MAS, have greatly helped the breeders to do so. However, if the breeders can now more easily identify good allele combinations, they are still limited by the possibility of actually combining them in the plant. Indeed, this depends on the number and localization of recombination events that occur between homologous chromosomes during meiosis, which ensures the reshuffling of genetic diversity for evolution and adaptation of species. This is especially true when crossing is done between different species where sequence divergence in homeologous regions can impair cross-over formation during meiosis (Canady et al. 2006). Mastering the recombination at the crossing stage would, therefore, be a wonderful tool to control the combination of the chromosomal regions that contain the favorable alleles while avoiding the presence of unfavorable alleles that can be linked to them. A key step of meiotic recombination is the formation of double strand breaks (DSBs) into the chromosomes that subsequently define loci of mutual genetic exchange. To manipulate meiotic recombination in crop plants, one could consider targeting the meiotic double strand breaks to specific regions to induce site-specific cross-over, where genetic exchange is needed (Wijnker and de Jong 2008). The SPO11 protein and its partners are the key actors of the meiotic-programmed DSBs. In yeast, it has been shown that a fusion of the SPO11 protein to the DNA-binding domain of the transcriptional activator Gal4 can stimulate DSB formation, with associated recombination, near Gal4-consensus-binding sites (Peciña et al. 2002). Based on these data, it would be tempting to test in plants whether SPO11 fusions with a variety of different DNA-binding domains, such as TALE effectors or dead CAS9 associated to specific sgRNA, could target DSBs to specific regions of the genome. Such a tool would be very useful to breeders to accelerate the combination of favorable alleles.

## Conclusion

Plant evolution relies on spontaneous genome mutations potentially resulting in new traits fixed by natural selection. Plant breeding also relies on natural genetic variability but, in addition, breeders have increased it using random mutagenesis. Genome editing now provides means to introduce almost any type of mutation and chromosome rearrangements in a very precise way. This not only empowers the breeders to accelerate and direct crop selection in an unprecedented way; it also opens up the door to an almost unlimited range of possibilities in terms of the combination of new alleles by erasing sexual barriers. These new tools could be integrated in breeding schemes very rapidly in the upcoming years. From a scientific point of view, the main limiting factor is the reliable and efficient identification of the genes underlying traits of interest and the evaluation of their combination on the value of these traits. For that matter, developing efficient gene-function analysis tools and precise high-throughput phenotyping methods are essential. However, the main uncertainty on the future use of these techniques for plant breeding is the regulatory framework that will be applied to their commercial products. As discussed in chapter 10, whether these plants and their products are considered under the GMO legislation and risk assessed as GMOs, or whether they are submitted to an alleviated legal framework or are completely deregulated, will have a profound impact on the development and the use of these techniques in plant breeding. The costs and delays associated to the GMO approval process would probably block the use of these techniques for most crops and traits, and would make it impossible for small breeders and seed companies to engage in the development of new varieties using genome editing, as it has already happened with conventional GMOs.

### **Author contribution statement**

All authors wrote and approved the final manuscript.
